# Subcutaneous Ticks in Wild Carnivores: Any Host-Related Differences?

**DOI:** 10.3390/ani12233411

**Published:** 2022-12-04

**Authors:** Barbara Moroni, Fabrizio Coenda, Aitor Garcia-Vozmediano, Arturo Nicoletti, Paola Pregel, Alessandra Mina, Laura Tomassone, Luca Rossi, Frine Eleonora Scaglione

**Affiliations:** 1 Istituto Zooprofilattico Sperimentale del Piemonte, Liguria e Valle d’Aosta, Via Bologna 148, 10154 Torino, Italy; 2 Department of Veterinary Sciences, University of Turin, Largo Braccini 2, 10095 Grugliasco, Italy

**Keywords:** tick, ectoparasite, red fox, wolves, *Ixodes* spp., wildlife, Italy

## Abstract

**Simple Summary:**

Ticks are obligate parasites living part of their life attached on the skin surface of different mammal species. In the last decades, there have been several reports of ticks found dead underneath the skin of foxes, raccoon dogs, golden jackals, domestic dogs, and a human being. The biological reasons behind this phenomenon are still unclear, although most of the reports are in canid species, suggesting that the immunological response of canids might favor it. The aim of this study was to investigate the presence of ticks under the skin of different wild carnivore species in Northwestern Italy, where they have never been described before. Out of 63 wild carnivores, 11 foxes were found infested with 51 dead ticks under the skin of the animals. All the preserved ticks collected underneath the skin of the foxes were identified as *Ixodes* spp., meaning that this tick species might be more frequently involved in the phenomenon, as already suggested by the scientific community. By contrast, no subcutaneous ticks were found in wolves, the other most prevalent wild canid species in Northwestern Italy, supporting the idea that the immune reaction of wolves may not favor the embedment of ticks underneath the skin.

**Abstract:**

Ticks under the skin have been shown in different canid species such as red fox, domestic dog, and raccoon dog. Despite being increasingly reported in Europe in the last decade, the biological mechanisms associated to subcutaneous ticks (SCT), as well as the predisposing factors, are not yet clear. The main goal of this study was to investigate the presence of SCT in wild carnivores in Northwestern Italy. Sixty-three wild carnivores were examined, and SCT were submitted to histological examination or stored in ethanol for morphological and molecular identification. A portion of the *cox1* gene and 16S rDNA were amplified, and positive PCR products were sequenced. Fifty-one small brown-coloured nodules of about 2 × 3 mm containing ticks in different decomposition stages were observed in 11 out of 30 foxes. Seven ticks were classified as *Ixodes ricinus*, while 14 ticks were determined only at the genus level (*Ixodes* spp.), and in two ticks no morphological key was applicable due to the advanced degradation status. By PCR, the rDNA fragment of six ticks (26.1%, 95% CI: 12.6–46.5%) was amplified, and BLAST analysis revealed a 99–100% nucleotide similarity to *I. ricinus*. At the histological examination, the inflammatory response varied from a mild to a moderate mixed infiltrate, primarily composed by neutrophils and lymphocytes. The results of this study confirm foxes as the main wild reservoir for SCT. The absence of SCT in other carnivores (badgers and martens) is in accordance with other studies. *Ixodes ricinus* is the most frequently reported tick species, corroborating the idea that longirostral ticks might be more frequently associated to SC embedment than brevirostral ticks.

## 1. Introduction

Hard ticks (fam. Ixodidae), either attached and engorging or crawling on the animal fur, are common findings in a wide range of mammals in Europe [1]. Carnivores are no exception, and some species, usually the most abundant and/or easily available for necropsies, such as the red fox (*Vulpes vulpes*), are now recognized as playing a remarkable role in the biology of several ticks and in the epidemiology of the associated tick-borne diseases [2,3].

As is known, ticks, having acquired a site for feeding, firmly anchor themselves to the host’s external skin through the rostrum to take a blood meal that, in adult females, may last for up to two weeks to complete engorgement [4,5]. Upon completion of the feeding process, ticks drop off the host to moult or lay eggs according to instar. Usually, tick bites cause mild to moderate, superficial, and transitory damage to the host skin, in the form of focal dermatitis at the attachment site, which is eventually itchy and can lead to secondary bacterial infection [6,7]. Nonetheless, a growing amount of recent literature has reported on the finding of hard ticks at various stages of degradation in the subcutis of red foxes in Germany, Czech Republic, Poland, Romania, and Sweden [8,9,10,11]. In these surveys, prevalence of subcutaneous ticks (SCT hereafter) ranged between 15.4 and 88.1%, suggesting common occurrence of this previously neglected phenomenon [8,9,11]. Three additional SCT cases were reported in raccoon dogs (*Nyctereutes procyonoides*) in Poland [12] and single cases in the golden jackal (*Canis aureus*) [10] and domestic dog (*Canis familiaris*) in Romania and Sweden, respectively [8,13]. No SCT were found in the grey wolf (*Canis lupus*) and other, non-canid, hosts, including members of the Felidae and Mustelidae families in Romania [10], but the sample size was quite limited in the case of the wolf and the Eurasian lynx (*Lynx lynx*). The reasons for possible host-related differences in the occurrence of SCT within carnivore communities are still unknown [10].

*Ixodes ricinus* ticks have been mainly involved in this phenomenon, but also other tick species including *I. canisuga*, *I. crenulatus*, *I. hexagonus*, and *Dermacentor reticulatus* have been previously reported (reviewed in [10]). The precise morphological identification of species, however, may be hindered in the case of SCT given their state of degradation [8,9]. Nevertheless, molecular methods have successfully led to determining the proper identification, although in relatively low percentages, of extended degraded ticks [11].

Recent histopathological investigation has provided a comprehensive description of the reactive cellular processes associated with the occurrence of SCT [8]. Main microscopic findings consisted of a cystic tick associated with a granulomatous panniculitis, both embedded in a well-developed fibrotic capsule. Inflammatory cells surrounded the tick, with a predominance of macrophages, epithelioid and multinucleated giant cells, neutrophils, and sparse eosinophils. By contrast, a mixture of cell debris, cholesterol crystal clefts, and macrophages dominated in the inner part of the SCT.

The main goal of this study was to investigate the presence and prevalence of SCT amongst wild carnivore species in northern Italy. In particular, we expanded SCT sampling in the so far poorly investigated grey wolf, by taking advantage of its favorable demographic trend in the region and the associated flow of wolf carcasses for dispersal, mortality, and epidemiological studies [14,15,16]. We also investigated the diversity of SCT by means of molecular tools in addition to morphology, and provided a description of histopathological findings around the location of SCT in a sample of infested red foxes.

## 2. Materials and Methods

Between February 2019 and December 2020, 63 wild carnivores from the Piedmont region, northern Italy, were examined at the Department of Veterinary Science of the University of Turin. Foxes were obtained through legal hunting activity according to Italian law, while wolves, badgers and martens were found dead and examined to determine the underlying cause (Table 1). All the available information (species, age, sex, sampling location) on these animals was recorded in a shared database (Table S1).

Carcasses were classified into three age classes based on body mass and tooth wear [17]: juvenile (<1 year-old); subadult (yearling); and adult (≥2 years-old).

All carcasses were skinned following a procedure including five main incisions: the first one was made from the mandibular symphysis to the ischiopubic region following the *linea alba*, cutting laterally to the penis if the animal was a male. The other four incisions were made on the inner medial faces of the limbs, starting from the metacarpals and the metatarsals, up to the reunion with the central cut.

Following these incisions, skin was separated from the corpse to detect the presence of SCT. Their localization was recorded according to Haut et al. [9], dividing the coat in ten different areas (ears, neck, axillar region, shoulder, forelimbs, back, belly, inguinal region, tail, hindlimbs).

Sarcoptic mange lesions were also recorded, and confirmed by microscopic identification of *Sarcoptes* mites.

Tissue samples for histological examination were fixed in 10% neutral buffered formalin (pH 7), wax embedded, and sectioned at 4 μm using a microtome (Leica Microsystems, Wetzlar, Germany). Slices were stained with haematoxylin and eosin and Masson’s trichrome, and examined by light microscopy.

A sample of SCT (*n* = 23 from seven foxes) was stored in 70% ethanol at 4 °C for morphological and molecular analysis. Tick identification was performed under a stereomicroscope following morphological criteria [18,19]. Moreover, DNA of individual SCT was extracted by using DNAzol reagent^®^ (Life Technologies LTD, Warrington, UK), according to the manufacturer’s instructions. Prior to DNA extraction, ticks were cut apart with sterile scalpel blades and immersed in lysis buffer (NaCl 0.1 M, Tris-HCl 0.21 M, pH 8 EDTA 0.05 M, SDS 0.5%) with proteinase-K (20 mg/mL) at 56 °C overnight.

For molecular identification, we targeted a portion of the *cox1* gene (732 bp) and of the 16S rDNA (455 bp) as previously described [20,21]. To minimize contamination and false-positive samples, the DNA extraction, PCR mix preparation, sample addition, and PCR analyses were performed in separate laboratories. Positive controls and negative water controls were used on every PCR assay performed in this study. Positive PCR products were purified using ExoSAP-IT PCR Clean-up Kit (GE Healthcare Limited, Chalfont, UK) and sent to an external service (BMR Genomics, Padua, Italy) for automatic sequencing. The sequences were analyzed with Bioedit [22] and then compared with reference sequences deposited in GenBank by using BLAST. Descriptive data were analysed using GraphPad InStat (version 9.00, GraphPad Software, La Jolla, CA, USA). Chi-squared test was applied to evaluate the association of SCT with the different coat areas. Association with sex, season and the coinfection with sarcoptic mange was explored using Fisher’s exact test. Associations were considered statistically significant when *p* < 0.05.

## 3. Results

**Figure 1:**
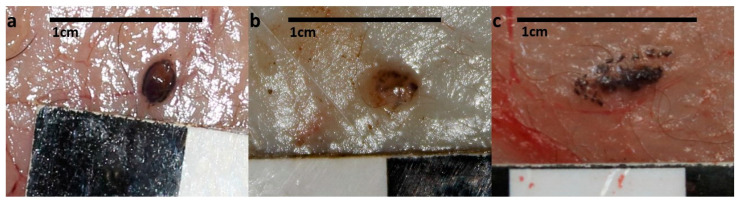
Macroscopic view of ticks with subcutaneous localization at various degradation stages. Well preserved *I. ricinus* in dorsal position with intact idiosoma (**a**), tick in early decomposition, the body is partially preserved, although not identifiable, and the appendix are not present (**b**), tick in advanced decomposition stage, with fragmented particles of the body (**c**).

Fifty-one small brown-coloured nodules of about 2 × 3 mm, apparently containing ticks in different decomposition stages, were observed in the hypodermis of 11 out of 30 foxes (36.7%, 95% CI: 21.9–54.5%) (Table 2; Figure 1). At the necropsy, the cause of death of wolves, badgers, pine and stone martens was attributed to polytrauma, while all hunted foxes died of gun-related haemorrhages (Table S1). All foxes, badgers and martens were adults, while 12 wolves were juveniles, 5 were sub-adults and 7 were adults.

Mean abundance of subcutaneous ticks in foxes was 1.7 ± 0.65. The number of SCT per examined fox ranged between 0 and 15. Eight foxes were infested with less than 5 subcutaneous ticks, and three of them with 9, 10 and 15 ticks. Thirty-five SCT were localized in the thoraco-abdominal region of the body, while the other 16 were localized in the head region and limbs (Table 2). Details on the distribution of SCT in red foxes were summarized in Table S1. No SCT were found in the remaining carnivore hosts.

The presence/absence of SCT was significantly associated with the thoraco-abdominal region (Pearson’s Chi-squared test, *p* < 0.001) but no association with sex, season, and mange coinfection was found. No hyperaemia nor other macroscopic alterations were detected around the localization of SCT. Sarcoptic mange was reported in four foxes, among which two presented SCT.

Seven SCT could be morphologically classified at the species level (*Ixodes ricinus*), and another 14 at the genus level (*Ixodes* spp; Table 2). In two ticks, the advanced degradation impeded any morphological approach. Twelve of twenty-one *Ixodes* ticks could be identified as adult females, while the instar could not be determined in the remaining specimens.

By PCR, we could amplify the rDNA fragment of six SCT, and BLAST analysis revealed a 99–100% nucleotide similarity to *I. ricinus* from Spain (GenBank Accession number: MH645517), Sweden (KX384810), Slovakia (MN947215, GU074589) and Poland (MK671574-88). All the sequences were deposited in GenBank database under the OP622941-4. No amplicons were obtained in the *cox1* PCR.

**Figure 2:**
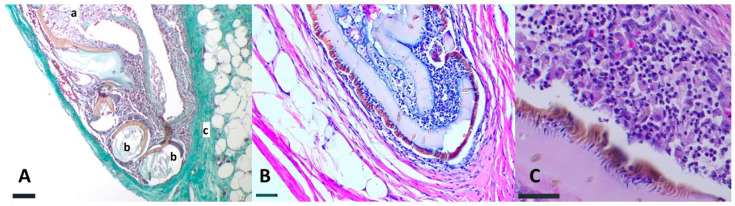
Histological sections of ticks in the SC tissue of red foxes from Italy. (**A**): moderate mixed infiltrate composed by neutrophils, lymphocytes and rare eosinophils surrounding the tick (a). Fragments of tick exoskeleton surrounded by inflammatory cells (b) and a complete multilayer connective tissue capsule surrounding the tick body (c) (Masson’s trichrome, bar 500 µm). (**B**): The inner part of the tick was composed of cell debris and a multilayer connective tissue capsule surrounding the tick body (HE, bar 300 µm). (**C**): mild mixed infiltrate surrounding the tick, and a thin connective tissue capsule (HE, 150 µm).

Histologically, embedded ticks were localized in the subcutaneous fat tissue and under the muscular layer of the subcutis (*panniculus carnosus*). The exoskeleton was mostly intact, and the appendages variably preserved, though not assignable to legs or palpi due to deformation of the tick. The inflammatory response varied from a mild to moderate mixed infiltrate surrounding the tick, primarily composed of neutrophils, lymphocytes and rare eosinophils (Figure 2).

Moreover, a complete multilayer connective tissue capsule surrounded the parasites (Figure 2B). The inner part of the tick was composed of cell debris (Figure 2A,B). No signs of extravasated erythrocytes, cholesterol crystal clefts, or blood degradation products were observed. The overlying skin did not show fibrosis or other alterations.

## 4. Discussion

Our results confirm foxes as the main wild species hosting SCT [9,10]. Their absence in non-canid carnivores (badgers and martens) is in line with the study of Mechouk et al. [10], in which no SCT were found in wild felids (*n* = 29) nor in mustelids (*n* = 30). In the same study, grey wolves were investigated for the presence of SCT for the first time, although the number of carcasses was low (*n* = 6). The absence of SCT in wolves in our study substantiates those findings and might suggest that wolves, unlike foxes, are poorly susceptible to tick embedment in the SC tissue.

*Ixodes ricinus* is the most frequently reported species, as in other studies of SCT in wildlife [8,9,10,13]. This finding corroborates the hypothesis that longirostral ticks have higher predisposition, compared to brevirostral ticks, to embedment in the SC tissue [8,9,10]. Moreover, the longer time spent on the host by adult female ticks compared to immature instars (larvae and nymphs) or adult males might favor the skin penetration process, as suggested by D’Amico et al. [8]. In our study, no males nor immature stages were identified in the SC.

The unsuccessful DNA amplification in the majority of analyzed SCT, despite using two different universal markers (*cox1* gene and *16S* rRNA gene), was likely due to the advanced degradation of collected specimens, and their organs and cellular structures. These results are in accordance with recent studies in foxes [9,11], which report no DNA amplification out of 902 ticks, or a low yield PCR products (22 out of 64 ticks; 34.4%). New proteomic techniques not relying on the amplification of DNA such as MALDI-TOF mass spectrometry should be considered to allow identification of degraded ticks at the species level [23,24,25].

All SCT identified in foxes were *Ixodes* spp., and *I. ricinus* could be identified in a representative subsample. *Ixodes ricinus* is the most abundant tick in wooded areas in northern Italy [26], even found at mountain altitudes (>1500 m) [27], and is considered one of the most common tick species found on red foxes countrywide, although several other taxa have been recorded [18,28].

Localization of SCT in the fur was systematically investigated in a single previous study [9], showing that ears, axillae and the inguinal area were the most frequent localization in red foxes. Accordingly, in our study, SCT were significantly associated with the thoraco-abdominal region of the body (including axillar region, shoulder, back, belly, inguinal region) of red foxes, while only few SCT were found on the head region (8/51).

Most of the SCT in the present study were surrounded by a mild degree of inflammation (granulomatous panniculitis), in agreement with previous studies [8,9]. The obvious degradation of those ticks and the mild degree of inflammation would be suggestive of a relatively long time interval elapsed since tick embedment in the subcutis. However, while in one study [9] the inflammatory response appeared related to tick degradation status, we could not demonstrate such association. In fact, a moderate inflammation was observed not only around ticks showing mild deformation or fragmentation of the exoskeleton, but also around ticks in advanced states of decomposition.

Despite being increasingly described in foxes in Europe, the actual biological and evolutionary reasons behind tick embedment in the subcutis of mammals, the host species association, and the frequency/geographical extent of this phenomenon, are not clear. Dwużnik et al. [11] advanced the hypothesis of a mechanism generated by the host-hyperimmune response during the first days after the tick attachment, leading to a deep penetration of the hypostome through the hypodermic layers, and its progressive embedment (also called “sinking”) underneath the skin, impeding it to resurface. As already shown for other ectoparasites, such as the mite *Sarcoptes scabiei* causing sarcoptic mange, different host species can have different immune responses [29,30,31,32] with various degrees of clinical signs depending on the age, sex, and body condition, as it may happen with ticks. In particular, red foxes would be less effective at countering sarcoptic mange (frequently showing severe skin lesions) due to a lack of memory T-cells after the contact with *Sarcoptes* mites [29,33], which might also explain the higher incidence of SCT in this species.

## 5. Conclusions

Despite the sample size of wolves in our study being still relatively limited, though the highest in literature so far, the fact that no wolves presented SCT, and that among other canids, only five additional reports of SCT ticks were made in three raccoon dogs in Poland [12], a domestic dog in Sweden [13] and a golden jackal in Romania [10], support the hypothesis that the immune reaction of these canids in the early phases of tick insertion may be less intense than that activated by foxes, thus preventing the embedment process underneath the skin.

Further studies are needed to identify the underlying biological reasons leading to the embedment process of ticks in the SC tissue, and to understand the pathogenesis and host-related immune response associated to this phenomenon.

## Figures and Tables

**Table 1:** Animal species examined in the present study and their infestation with SC ticks.

Host Species	N	Animals Found with SCT
Red fox (*Vulpes vulpes*)	30	11
Wolf (*Canis lupus*)	24	0
Badger (*Meles meles*)	6	0
Stone marten (*Martes foina*)	2	0
Pine marten (*Martes martes*)	1	0

**Table 2:** Red foxes found positive for SCT. Body localization is intended as the coat area where ticks were found in the subcutis, namely the head region (including ears and neck), the thoraco-abdominal region (including axillar region, shoulder, back, belly, inguinal region) and limbs (including, tail, forelimbs, hindlimbs). Morphological identification has been performed under stereomicroscope following morphological criteria, while molecular ID refers to the outcome of *cox1* gene and 16S rDNA amplification (see Method section).

ID	Sex	No. of SCT	SCT Morphological ID	SCT Molecular ID	SCT Localization			Mange
					Head Region	Thoraco-Abdominal Region	Limbs	
37	M	1	*Ixodes* spp.	nd	1	0	0	no
98	F	1	*Ixodes* spp.	nd	1	0	0	no
129	F	15	*I. ricinus* (3), *Ixodes* spp. (12)	*I. ricinus*	2	10	3	no
132	F	10	*I.ricinus* (2), *Ixodes* spp. (8)	*I. ricinus*	0	8	2	no
133	M	5	*I. ricinus* (1), *Ixodes* spp. (4)	*I. ricinus*	0	4	1	no
145	M	4	*Ixodes* spp.	*I. ricinus*	0	4	0	yes
V_1	M	2	*I. ricinus* (1), *Ixodes* spp. (1)	*I. ricinus*	0	0	2	no
75	F	1	*Ixodes* spp.	nd	1	0	0	no
87	M	2	*Ixodes* spp.	nd	2	0	0	yes
V_14	M	1	*Ixodes* spp.	nd	1	0	0	no
V_18	F	9	*Ixodes* spp.	*I. ricinus*	0	9	0	no

Note: ‘nd’ = tick species could not be ascertained.

## Data Availability

All relevant data are provided in the present study or in Supplementary Materials.

## Supplementary Materials

The following are available online at https://www.mdpi.com/article/10.3390/ani12233411/s1, Table S1: Sampled animals, corporal distribution and abundance of SCT in red foxes, Piedmont, 2019–2020.

## References

[1] Kolonin G.V. (2007). Mammals as hosts of Ixodid ticks (Acarina, Ixodidae). Entomol. Rev..

[2] Hofmeester T.R., Jansen P.A., Wijnen H.J., Coipan E.C., Fonville M., Prins H.H.T., Sprong H., Wieren S.E. (2017). Van Cascading effects of predator activity on tick-borne disease risk. Proc. R. Soc. B Biol. Sci..

[3] Lesiczka P.M., Rudenko N., Golovchenko M., Juránková J., Daněk O., Modrý D., Hrazdilová K. (2022). Red fox (*Vulpes vulpes*) play an important role in the propagation of tick-borne pathogens. Ticks Tick. Borne. Dis..

[4] Richter D., Matuschka F.R., Spielman A., Mahadevan L. (2013). How ticks get under your skin: Insertion mechanics of the feeding apparatus of *Ixodes ricinus* ticks. Proc. R. Soc. B Biol. Sci..

[5] Oliver J.H. (1989). Biology and systematics of ticks (Acari: Ixodida). Annu. Rev. Ecol. Syst..

[6] Wikel S. (2013). Ticks and tick-borne pathogens at the cutaneous interface: Host defenses, tick countermeasures, and a suitable environment for pathogen establishment. Front. Microbiol..

[7] Cross C.E., Stokes J.V., Alugubelly N., Ross A.M.L., Willeford B.V., Walker J.D., Varela-Stokes A.S. (2022). Skin in the game: An assay to monitor leukocyte infiltration in dermal lesions of a guinea pig model for tick-borne rickettsiosis. Pathogens.

[8] D’Amico G., Juránková J., Tăbăran F.A., Frgelecová L., Forejtek P., Matei I.A., Ionică A.M., Hodžić A., Modrý D., Mihalca A.D. (2017). Occurrence of ticks in the subcutaneous tissue of red foxes, *Vulpes vulpes* in Czech Republic and Romania. Ticks Tick. Borne. Dis..

[9] Haut M., Król N., Obiegala A., Seeger J., Pfeffer M. (2020). Under the skin: Ixodes ticks in the subcutaneous tissue of red foxes (*Vulpes vulpes*) from Germany. Parasit. Vectors.

[10] Mechouk N., Deak G., Ionică A.M., Ionescu D.T., Chișamera G.B., Gherman C.M., Mihalca A.D. (2021). Subcutaneous ticks: A first report in a golden jackal, and their absence in non-canid carnivores. Parasit. Vectors.

[11] Dwużnik D., Mierzejewska E.J., Kowalec M., Alsarraf M., Stańczak Ł., Opalińska P., Krokowska-Paluszak M., Górecki G., Bajer A. (2020). Ectoparasites of red foxes (*Vulpes vulpes*) with a particular focus on ticks in subcutaneous tissues. Parasitology.

[12] Matysiak A., Wasielewski O., Wlodarek J., Ondrejkova A., Tryjanowski P. (2018). First report of ticks in the subcutaneous tissue of the raccoon dog *Nyctereutes procyonoides*. Vet. Med..

[13] Christensson D., Zakrisson G. (2010). Others Ticks, *Ixodes ricinus* in the sub-cutaneous tissues of a dog and foxes. Sven. Veterinärtidning.

[14] Moroni B., Rossi L., Meneguz P.G., Orusa R., Zoppi S., Robetto S., Marucco F., Tizzani P. (2020). *Dirofilaria immitis* in wolves recolonizing northern Italy: Are wolves competent hosts?. Parasites Vectors.

[15] Marucco F., Pilgrim K.L., Avanzinelli E., Schwartz M.K., Rossi L. (2022). Wolf Dispersal Patterns in the Italian Alps and Implications for Wildlife Diseases Spreading. Animals.

[16] Bezerra-Santos M.A., Moroni B., Mendoza-Roldan J.A., Perrucci S., Cavicchio P., Cordon R., Cianfanelli C., Lia R.P., Rossi L., Otranto D. (2022). Wild carnivores and *Thelazia callipaeda* zoonotic eyeworms: A focus on wolves. Int. J. Parasitol. Parasites Wildl..

[17] Gipson P., Ballard W.B., Nowak R.M., Mech D.L. (2000). Accuracy and Precision of Estimating Age of Gray Wolves by Tooth Wear. J. Wildl. Manag..

[18] Cringoli G., Iori A., Rinaldi L., Veneziano V., Genchi C. (2005). Zecche. Mappe Parassitol..

[19] Estrada-Peña A., Mihalca A.D., Petney T.N. (2018). Ticks of Europe and North Africa: A Guide to Species Identification.

[20] Chitimia L., Iustin R.L., Wu C.X. (2010). Genetic characterization of ticks from southwestern Romania by sequences of mitochondrial cox 1 and nad 5 genes. Exp. Appl. Acarol..

[21] D’Oliveira C., Van Der Weide M., Jacquiet P., Jongejan F. (1997). Detection of *Theileria annulata* by the PCR in ticks (Acari: Ixodidae) collected from cattle in Mauritania. Exp. Appl. Acarol..

[22] Hall T.A. (1999). BioEdit: A user-friendly biological sequence alignment editor and analysis program for Windows 95/98/NT. Nucleic Acids Symp. Ser..

[23] Yssouf A., Almeras L., Berenger J., Laroche M., Raoult D., Parola P. (2015). Ticks and Tick-borne Diseases Identification of tick species and disseminate pathogen using hemolymph by MALDI-TOF MS. Ticks Tick. Borne. Dis..

[24] Diarra A.Z., Almeras L., Laroche M., Berenger J., Bocoum Z., Dabo A., Doumbo O., Kone A.K., Raoult D., Parola P. (2017). Molecular and MALDI-TOF identification of ticks and tick-associated bacteria in Mali. PLoS Neglected Trop. Dis..

[25] Ahamada M’madi S., Diarra A.Z., Almeras L., Parola P. (2022). Identification of ticks from an old collection by MALDI-TOF MS. J. Proteomics.

[26] Zanet S., Ferroglio E., Battisti E., Tizzani P. (2020). Ecological niche modelling of *Babesia* spp. infection in wildlife experimentally evaluated in Northern Italy with reference to questing *Ixodes ricinus* ticks. Geospat. Health.

[27] Garcia-Vozmediano A., Krawczyk A.I., Sprong H., Rossi L., Ramassa E., Tomassone L. (2020). Ticks climb the mountains: Ixodid tick infestation and infection by tick-borne pathogens in the Western Alps. Ticks Tick. Borne. Dis..

[28] Perrucci S., Verin R., Mancianti F., Poli A. (2016). Sarcoptic mange and other ectoparasitic infections in a red fox (*Vulpes vulpes*) population from central Italy. PAREPI.

[29] Turchetto S., Obber F., Rossi L., D’Amelio S., Cavallero S., Poli A., Parisi F., Lanfranchi P., Ferrari N., Dellamaria D. (2020). Sarcoptic Mange in Wild Caprinae of the Alps: Could Pathology Help in Filling the Gaps in Knowledge?. Front. Vet. Sci..

[30] Valldeperes M., Moroni B., Rossi L., López-Olvera J.R., Velarde R., Molinar Min A.R., Mentaberre G., Serrano E., Angelone S., Lavín S. (2021). First report of interspecific transmission of sarcoptic mange from Iberian ibex to wild boar. Parasit. Vectors.

[31] Oleaga A., Casais R., Prieto J.M., Gortázar C., Balseiro A. (2012). Comparative pathological and immunohistochemical features of sarcoptic mange in five sympatric wildlife species in Northern Spain. Eur. J. Wildl. Res..

[32] Moroni B., Rossi L., Bernigaud C., Guillot J. (2022). Zoonotic Episodes of Scabies: A Global Overview. Pathogens.

[33] Astorga F., Carver S., Almberg E.S., Sousa G.R., Wingfield K., Niedringhaus K.D., Van Wick P., Rossi L., Xie Y., Cross P. (2018). International meeting on sarcoptic mange in wildlife, June 2018, Blacksburg, Virginia, USA. Parasites Vectors.

